# Association between sedentary behavior and depression among South Korean adolescents

**DOI:** 10.1186/s12888-022-04262-x

**Published:** 2022-09-21

**Authors:** Jinhyun Kim, Hyunkyu Kim, Sung-In Jang, Eun-Cheol Park

**Affiliations:** 1 Department of Preventive Medicine, Yonsei University College of Medicine, 50 Yonsei-ro, Seodaemun-gu, Seoul, 03722 Republic of Korea; 2 Institute of Health Services Research, Yonsei University, Seoul, Republic of Korea; 3 Department of Psychiatry, Yonsei University College of Medicine, Seoul, Republic of Korea

**Keywords:** Sedentary behavior, Depressed mood, Suicide, KYRBS

## Abstract

**Background:**

The symptoms and outcomes of depressed mood are considered severe social issues among Korean adolescents. However, it is difficult to detect depressed mood and evaluate the factors associated with suicide among such individuals. Identifying the risk factors of depressed mood would allow for improved perspectives for interventions. Therefore, in this study, we investigated the association between sedentary behavior and the prevalence of depressed mood.

**Methods:**

From 2014 to 2020, the Korea Youth Risk Behavior Survey (KYRBS), which is a web-based self-report survey, was used for analysis. A total of 366,405 individuals participated in this study. Sedentary behavior was divided into 3 groups based on the duration of sedentary behavior: low sedentary time group (LS, 25 percentile), middle sedentary time group (MS, from 25 to 75 percentile, reference), and high sedentary time group (HS, above 75 percentile). Further, sedentary behavior is divided into 4 subgroups based on weekdays or weekends and owing to studying or non-studying. The chi-square test and multivariate logistic regression were used in this study.

**Results:**

Compared to the MS, which is the reference, male participants in both the LS and HS had experienced depressed mood (adjusted odds ratio (OR): 1.035, 95% CI = 1.003-1.068 in the LS, adjusted OR: 1.091, CI = 1.055-1.129 in the HS). Among females, only the HS was statistically significant (adjusted OR: 1.039, 95% CI = 1.011-1.069 in HS). Korean adolescents with longer sedentary durations during weekdays regardless of the cause of sedentary behavior are positively associated with depressed mood with suicidality in the HS for both genders.

**Conclusion:**

This study found a positive association between the prevalence of depressed mood and sedentary behavior, and it focused on the cause and timing. Interventions targeting sedentary behavior could be effective in reducing depressed mood and suicidality among adolescents.

## Introduction

In South Korea, depression among adolescents is considered a concerning social issue. Depression is a significantly common mental illness among adolescents, and it can cause significant mental and physical sequelae. In 2020, 27.4% of high school students and 22.9% of middle school students reported that they felt depressed throughout the year [1]. The impulse to commit suicide, which is a symptom of depression, is at 3.9% among students [2]. Depression among adolescents can result in maladaptive outcomes, such as failure to graduate secondary school, low employment [3], substance abuse [4] and mental illness throughout adulthood [5]. Additionally, it can result in increased levels of physical illnesses, such as type 2 diabetes mellitus [6], obesity [7], and migraine headaches [8].

Early intervention is the key to preventing depressed mood or suicide. However, social stigma [9], different core depressive symptoms related to adult depressed mood [10], and the lack of social support make early recognition difficult. Therefore, identifying the predictors or risk factors of depression among adolescents is crucial to identifying students who are at a high risk of developing depression. For example, academic distress from competitive surroundings causes compromised quality of life [11] and increased suicidal ideation/behavior [12]. Similarly, the most concerning issue among Korean adolescents is studying and writing tests in a very competitive environment [1]. Additionally, conflictual parental relationships [13] and genetic predispositions [14] could also be contributing factors.

On the contrast, sedentary behavior has been considered a modifiable risk factor for depression in many recent studies. According to a UK-based prospective cohort study, a 1 h increase in sedentary time at the ages of 12, 14, and 16 is associated with a higher depression score at the age of 18 years [15]. In a US-based study, increased sedentary behavior and less break-time during sitting are associated with depressive moods and anxiety [16]. Most recent studies have focused on the relationships between total sedentary time, exercise, and the prevalence of depression or depressed mood. So, we analyzed not only the association between total sedentary time and prevalence of depressed mood, but also specific sedentary behavior based on day of week or cause of sedentary behavior. In detail, day of week was divided into 2 groups: weekdays and weekends, and causes were divided into 2 groups: studying and non-studying. Moreover, we also focused on the depressed mood which was divided into 2 groups: without suicidality and with suicidality. The hypothesis of this study is that the total sedentary time, especially due to studying which is the most concerning issue among Korean students [1], has a positive association with depressed mood.

## Methods

### Study population and data

The Korea Youth Risk Behavior Web-based Survey (KYRBS) conducted from 2014 to 2020 is the data source for this study. The period of data inclusion was determined based on the begin of survey questions about sedentary behavior. KYRBS is an annual, nation-wide, anonymous, self-report online survey conducted since 2005, and the Korea Disease Control and Prevention Agency (KDCA), Ministry of Education of Korea (MOE), and the Ministry of Health and Welfare of Korea (MOHW) conduct this survey. The participants of KYRBS involve students in 400 middle schools and 400 high schools. The purpose of KYRBS is the production of data regarding Korean students’ health conditions and the patterns of their daily lives.

### Measures

#### Depressed mood and Suicidality

Depressed mood was evaluated by questioning students whether they had felt sadness or despair that induced the interruption of daily life lasting for at least two weeks in the recent one year. In this study, suicidality involves suicidal ideation, suicidal plans, and suicidal attempts. The survey asked participants whether they had experienced suicidal ideation, made suicidal plans, or attempted suicide in the past one year. We classified depressive group into 2 categories: depressed mood without suicidality and depressed mood with suicidality.

#### Sedentary time

Participants provided the mean duration of sedentary behavior per day through a self-report. This question was divided into 4 items based on the day of the week and the cause of sitting. Specifically, weekday or weekend represented one criterion, and studying or non-studying represented the other. ‘Longer during weekday’ means students who had spent more sedentary time during weekdays than weekends. ‘Study ≥ Other causes’ means students who had spent more sedentary time due to studying (doing homework, attending a class, or using a computer for online lectures) than non-studying (watching television, playing computer games, resting, and chatting) regardless of either weekdays or weekends. Therefore, 4 independent variables were adopted for subgroup analysis of total sedentary time: Longer during weekday & Study < Other causes; Longer during weekday & Study ≥ Other causes; Longer during weekend & Study < Other causes; Longer during weekend & Study ≥ Other causes. The total average sedentary time per day was divided into three groups. Meanwhile, the participants whose sedentary time was below 6.4 h (25 percentile) represent the ‘low’ sedentary time group (LS), and those whose sedentary time was above 12.9 h (75 percentile) represent the ‘high’ sedentary time group (HS). The group of participants whose sedentary time lasted between 6.4 and 12.9 h was named the ‘middle’ sedentary time group (MS). Meanwhile, when participants answered that they had spent sedentary behavior more than 24 h, data were deleted.

#### Covariates

For adjustment of analysis, we included gender, age, type of family, economic status, grade, drinking, smoking, physical activity, perceived stress level, self-report health condition, average of sleep duration, and the ‘year 2020’ as covariates. The type of family was divided into 3 groups: living with both parents, one parent, and none. Economic status, grade, perceived stress level, and self-reported health conditions were also divided into 3 groups. Sleep duration was calculated using self-reported sleep induction time to self-reported wake up time. Sleep duration was divided into 2 groups based on an 8-h duration, which is the recommended sleep duration for teenagers [17]. Level of physical activity was divided into high and low groups. Students who met the physical activity recommendation were classified as high physical activity group. The recommendation was daily physical activity with moderate or vigorous intense (shortness of breath or sweating) and with more than 3 days of vigorous aerobic activity, with bone and muscle strengthening exercise [18, 19]. ‘Year 2020’ is one of the covariates because COVID-19 has affected people’s daily lives, including their emotional statuses and the prevalence of mental illness since 2020 [20, 21].

### Statistical analysis

For participants’ characteristics analysis, chi-square tests were used. To compare the relationship between depressed mood and sedentary behavior, we applied the multivariate logistic regression analysis with covariate adjustment. Subgroup analyses were conducted to establish the detailed effects of sedentary behavior on depressed mood. Additionally, depressed mood groups were classified into 2 groups, and the criterion for grouping was the experience of suicidality. All the data are presented as odds ratios (OR) and 95% confidence intervals (CI), and the analyses were conducted through stratified sampling variables (strata) and weighted variables. SAS software version 9.4 (SAS Institute, Cary, North Carolina, USA) was used for this study. Statistical significance was determined as p-value <0.05.

## Results

The results of the socioeconomic and health-related characteristics of the study population classified based on gender are presented in Table 1. The total number of participants was 366,405, including 184,514 males (50.4%) and 181,891 females (49.6%). 19.9% of males and 30.4% of females responded that they had experienced depressed mood for two weeks throughout the recent year. Participants belonging to the HS reported higher levels of depressed mood. Specifically, 18.8% of males in the LS, 19.4% in the MS, and 22.9% in the HS reported having experienced depressed mood. Additionally, 28.9% of female participants in the LS, 29.7% in the MS, and 32.6% in the HS reported having experienced depressed mood during the previous year. Further, a shorter sleep duration showed higher levels of depressive experiences than a longer one. Age, type of family, economic status, grade, drinking, smoking, physical activity, perceived stress level, self-report health condition, and COVID-19 period were identified as having statistical significance.

**Table 1:** Socioeconomic and health-related characteristics of participants according to the presence of depressed mood

Variables	Male (***N*** = 184,514, 50.4%)				***p***-value	Female (***N*** = 181,891, 49.6%)				***p***-value
	Depressive		Not-depressive			Depressive		Not-depressive		
	N	(%)	N	(%)		N	(%)	N	(%)	
**Total Sedentary time**					**<0.0001**					**<0.0001**
Low	10,944	(18.8)	47,278	(81.2)		9600	(28.9)	23,672	(71.1)	
Middle	17,402	(19.4)	72,250	(80.6)		28,714	(29.7)	67,807	(70.3)	
High	8385	(22.9)	28,255	(77.1)		16,972	(32.6)	35,126	(67.4)	
**Specific Sedentary time**					**<0.0001**					**<0.0001**
Longer during weekday	19,449	(19.4)	80,921	(80.6)		35,486	(29.6)	84,345	(70.4)	
Longer during weekend	17,282	(20.5)	66,862	(79.5)		19,800	(31.9)	42,260	(68.1)	
**Specific Sedentary time**					0.8200					**<0.0001**
Study < Other causes	17,006	(19.8)	68,786	(80.2)		20,344	(31.9)	43,493	(68.1)	
Study ≥ Other cause	19,725	(20.0)	78,997	(80.0)		34,942	(29.6)	83,112	(70.4)	
**Sleep Duration**					**<0.0001**					**<0.0001**
> 8 hours	8082	(15.2)	45,211	(84.8)		8889	(23.9)	28,308	(76.1)	
≤ 8 hours	28,649	(21.8)	102,572	(78.2)		46,397	(32.1)	98,297	(67.9)	
**COVID-19 period**					**0.0005**					**0.0029**
Before COVID-19 period	32,279	(20.0)	128,829	(80.0)		48,913	(30.5)	111,400	(69.5)	
After COVID-19 period	4452	(19.0)	18,954	(81.0)		6373	(29.5)	15,205	(70.5)	
**Age**					**<0.0001**					**<0.0001**
12-15	19,068	(18.0)	86,921	(82.0)		30,029	(28.6)	74,860	(71.4)	
16-18	17,663	(22.5)	60,862	(77.5)		25,257	(32.8)	51,745	(67.2)	
**Living with Parents**					**<0.0001**					**<0.0001**
Both Parents	30,999	(19.5)	127,740	(80.5)		48,889	(29.9)	114,404	(70.1)	
Single Parent	1907	(23.0)	6387	(77.0)		2831	(35.3)	5197	(64.7)	
None	3825	(21.9)	13,656	(78.1)		3566	(33.7)	7004	(66.3)	
**Economic Status**					**<0.0001**					**<0.0001**
Low	7313	(27.4)	19,408	(72.6)		11,146	(40.4)	16,459	(59.6)	
Middle	15,529	(18.5)	68,297	(81.5)		26,078	(28.7)	64,837	(71.3)	
High	13,889	(18.8)	60,078	(81.2)		18,062	(28.5)	45,309	(71.5)	
**Grade**					**<0.0001**					**<0.0001**
High	12,933	(17.7)	60,225	(82.3)		18,060	(26.2)	50,869	(73.8)	
Middle	9675	(18.7)	42,136	(81.3)		15,808	(28.8)	38,989	(71.2)	
Low	14,123	(23.7)	45,422	(76.3)		21,418	(36.8)	36,747	(63.2)	
**Alcohol Status**					**<0.0001**					**<0.0001**
Never	16,340	(15.6)	88,247	(84.4)		30,331	(25.4)	89,059	(74.6)	
Ever	20,391	(25.5)	59,536	(74.5)		24,955	(39.9)	37,546	(60.1)	
**Smoking Status**					**<0.0001**					**<0.0001**
Never	25,776	(17.6)	121,088	(82.4)		48,421	(28.8)	119,827	(71.2)	
Ever	10,955	(29.1)	26,695	(70.9)		6865	(50.3)	6778	(49.7)	
**Physical Activity**					**<0.0001**					**<0.0001**
Low	34,054	(19.7)	139,180	(80.3)		54,175	(30.3)	124,798	(69.7)	
High	2677	(23.7)	8603	(76.3)		1111	(38.1)	1807	(61.9)	
**Perceived Stress level**					**<0.0001**					**<0.0001**
Low	2664	(5.6)	44,834	(94.4)		1788	(7.0)	23,794	(93.0)	
Middle	12,397	(15.1)	69,776	(84.9)		14,632	(19.4)	60,819	(80.6)	
High	21,670	(39.5)	33,173	(60.5)		38,866	(48.1)	41,992	(51.9)	
**Self-Reported Health Status**					**<0.0001**					**<0.0001**
High	24,625	(17.4)	116,818	(82.6)		30,148	(24.9)	90,873	(75.1)	
Middle	8598	(25.6)	25,048	(74.4)		17,599	(37.6)	29,178	(62.4)	
Low	3508	(37.2)	5917	(62.8)		7539	(53.5)	6554	(46.5)	
**Total**	36,731	(19.9)	147,783	(80.1)		55,286	(30.4)	126,605	(69.6)	

Table 2 shows the results of multivariate logistic regression, which represents the analysis of the relationship between depressed mood and sedentary behavior using covariates. Relative to the MS, which is the reference group, male participants in both the LS and HS had experienced more depressed mood (adjusted OR: 1.035, 95% CI = 1.003-1.068 in the LS, adjusted OR: 1.091, CI = 1.055-1.129 in the HS). However, among the female participants, only the HS is statistically significant (adjusted OR: 1.039, 95% CI = 1.011-1.069 in HS). Long sleep duration, increased age, having both patents, middle and high economic status, high grade, no history of alcohol and smoking, low physical activity, low perceived stress level, and high self-report health status show a negative association with depressed mood, as shown in Table 2.

**Table 2:** The association between the total sedentary behavior and the prevalence of depressed mood

Variables	Male (***N*** = 184,514)		Female (***N*** = 181,891)	
	Depressive		Depressive	
	Adjusted OR	95% CI	Adjusted OR	95% CI
**Total Sedentary time**				
Low	1.035^*^	1.003-1.068	1.014	0.981-1.048
Middle	1.000		1.000	
High	1.091^*^	1.055-1.129	1.039^*^	1.011-1.069
**Sleep Duration**				
> 8 hours	1.000		1.000	
≤ 8 hours	1.193^*^	1.153-1.234	1.172^*^	1.134-1.212
**COVID-19 period**				
Before COVID-19 period	1.000		1.000	
After COVID-19 period	1.015	0.971-1.062	1.048^*^	1.005-1.093
**Age**				
12-15	1.000		1.000	
16-18	0.931^*^	0.903-0.960	0.839^*^	0.816-0.863
**Living with Parents**				
Both Parents	1.000		1.000	
Single Parent	1.057	0.992-1.127	1.022	0.962-1.085
None	1.146^*^	1.094-1.202	1.094^*^	1.037-1.154
**Economic Status**				
Low	1.000		1.000	
Middle	0.820^*^	0.789-0.852	0.815^*^	0.787-0.845
High	0.938^*^	0.901-0.977	0.940^*^	0.905-0.976
**Grade**				
High	1.000		1.000	
Middle	1.052^*^	1.017-1.089	1.127^*^	1.094-1.162
Low	1.183^*^	1.144-1.223	1.340^*^	1.301-1.381
**Alcohol Status**				
Never	1.000		1.000	
Ever	1.450^*^	1.408-1.494	1.538^*^	1.497-1.581
**Smoking Status**				
Never	1.000		1.000	
Ever	1.439^*^	1.389-1.491	1.591^*^	1.520-1.665
**Physical Activity**				
Low	1.000		1.000	
High	1.397^*^	1.324-1.475	1.462^*^	1.333-1.603
**Perceived Stress level**				
Low	1.000		1.000	
Middle	2.797^*^	2.668-2.932	2.949^*^	2.792-3.116
High	9.414^*^	8.984-9.865	9.797^*^	9.275-10.348
**Self-Reported Health Status**				
High	1.000		1.000	
Middle	1.207^*^	1.168-1.248	1.348^*^	1.312-1.384
Low	1.629^*^	1.546-1.716	1.991^*^	1.910-2.075

Abbreviations: *OR* Odds ratio, *CI* Confidence intervals, *COVID-19* Coronavirus Disease 2019

^*^Statistically Significant

The subgroup analyses of sedentary behavior are shown in Table 3. After covariate adjustment, participants who spent more sedentary time during weekdays and due to non-studying show a positive association with statistical significance in not only the HS for both genders but also the LS among males (adjusted OR: 1.119, 95% CI = 1.023-1.223 in LS male, adjusted OR: 1.114, 95% CI = 1.024-1.212 in HS male, adjusted OR: 1.111, 95% CI = 1.035-1.191 in HS female). Low levels of sedentary behavior during weekends and due to non-studying are negatively associated with depressed mood among females (adjusted OR: 0.895, 95% CI = 0.827-0.968 in LS). Among male students whose sedentary behavior lasted longer during weekdays and due to studying, only the high sedentary time groups were positively associated with depressed mood (adjusted OR: 1.148, 95% CI = 1.055-1.249 in male).

**Table 3:** Analysis of the association between specific sedentary behavior and the prevalence of depressed mood

Variables	Male					Female				
	Low		Middle	High		Low		Middle	High	
	Adjusted OR	95% CI	Adjusted OR	Adjusted OR	95% CI	Adjusted OR	95% CI	Adjusted OR	Adjusted OR	95% CI
**Subtypes of Sedentary behavior**										
Longer during weekday & Study < Other causes	1.111^*^	1.022-1.207	1.000	1.137^*^	1.040-1.242	1.088	1.002-1.180	1.000	1.119^*^	1.040-1.203
Longer during weekday & Study ≥ Other causes	0.969	0.916-1.026	1.000	1.122^*^	1.065-1.182	0.983	0.938-1.031	1.000	1.075^*^	1.033-1.119
Longer during weekend & Study < Other causes	0.972	0.917-1.030	1.000	0.985	0.925-1.050	0.895	0.827-0.968^*^	1.000	0.994	0.938-1.054
Longer during weekend & Study ≥ Other causes	0.997	0.919-1.081	1.000	1.148^*^	1.055-1.249	0.941	0.863-1.027	1.000	1.019	0.946-1.096
**COVID-19 period**										
Before COVID-19 period	1.033	0.999-1.068	1.000	1.093^*^	1.054-1.134	1.024	0.989-1.060	1.000	1.045^*^	1.015-1.077
After COVID-19 period	1.057	0.962-1.162	1.000	1.076	0.984-1.177	0.923	0.827-1.030	1.000	1.006	0.928-1.090
**Sleep Duration**										
> 8 hours	1.043	0.982-1.107	1.000	1.176^*^	1.078-1.282	0.909^*^	0.848-0.974	1.000	1.023	0.945-1.106
≤ 8 hours	1.036	0.998-1.075	1.000	1.078^*^	1.039-1.119	1.047^*^	1.009-1.087	1.000	1.045^*^	1.014-1.076
**Grade**										
High	1.114^*^	1.055-1.177	1.000	1.138^*^	1.079-1.202	1.039	0.975-1.106	1.000	1.026	0.981-1.073
Middle	1.005	0.946-1.068	1.000	1.078^*^	1.009-1.152	0.995	0.937-1.056	1.000	1.016	0.967-1.069
Low	0.993	0.943-1.046	1.000	1.031	0.969-1.096	1.011	0.960-1.065	1.000	1.075^*^	1.024-1.129
**Physical Activity**										
Low	1.041^*^	1.008-1.076	1.000	1.093^*^	1.055-1.132	1.018	0.985-1.053	1.000	1.040*	1.011-1.053
High	0.969	0.862-1.090	1.000	1.079	0.926-1.258	0.859	0.696-1.059	1.000	1.007	0.777-1.304

Adjusted for Age, Living with Parents, Economic status, Grade, Alcohol status, Smoking status, Physical activity, Perceived stress level, Self-report Health status, Sleep duration, year 2020(Coronavirus Disease 2019)

Abbreviations: *OR* Odds ratio, *CI* Confidence intervals

^*^Statistically significant

**Fig. 1:**
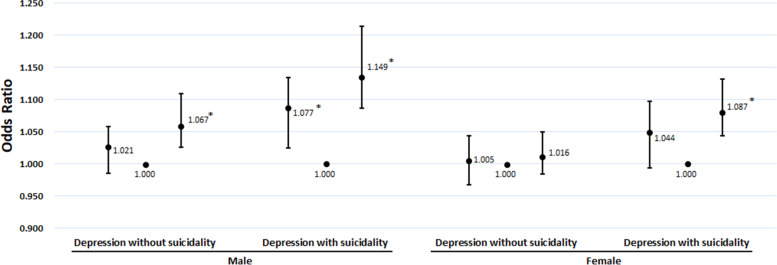
The association between the sedentary behavior and the depressed mood with and without suicidality. Adjusted for Age, Living with Parents, Economic status, Grade, Alcohol status, Smoking status, Physical activity, Perceived stress level, Self-report Health status, Sleep duration, year 2020(Coronavirus Disease 2019). *: Statistically significant

Dependent subgroup analyses, which are divided into the existence of suicidality, are presented in Fig. 1. Participants in the LS are only statistically significant in depressed mood with suicidality among males. However, participants in the HS are positively associated with depressed mood regardless of suicidality, except for depressed mood without suicidality among females.

The association between specific sedentary behavior and depressed mood with and without suicidality are shown in Fig. 1 and Table 4. Participants in HS and in longer during weekday are positively associated with depressed mood with suicidality than the MS regardless of the cause of sedentary behavior.

**Table 4:** Analysis of the association between specific sedentary behavior and the prevalence of depressed mood with and without suicidality

Variables		Male					Female				
		Low		Middle	High		Low		Middle	High	
		Adjusted OR	95% CI	Adjusted OR	Adjusted OR	95% CI	Adjusted OR	95% CI	Adjusted OR	Adjusted OR	95% CI
**Depressed mood without Suicidality**	**Specific Sedentary behavior**										
	Longer during weekday & Study < Other causes	1.106^*^	1.005-1.217	1.000	1.095	0.987-1.214	1.078	0.984-1.181	1.000	1.081	0.995-1.174
	Longer during weekday & Study ≥ Other causes	0.964	0.904-1.027	1.000	1.089^*^	1.026-1.156	1.001	0.950-1.056	1.000	1.057^*^	1.010-1.106
	Longer during weekend & Study < Other causes	0.986	0.922-1.055	1.000	0.982	0.914-1.055	0.857^*^	0.781-0.940	1.000	0.972	0.908-1.039
	Longer during weekend & Study ≥ Other causes	1.008	0.918-1.107	1.000	1.122^*^	1.018-1.237	0.965	0.875-1.065	1.000	0.997	0.917-1.084
**Depressed mood with Suicidality**	**Specific Sedentary behavior**										
	Longer during weekday & Study < Other causes	1.122	0.980-1.285	1.000	1.255^*^	1.091-1.444	1.104	0.972-1.252	1.000	1.173^*^	1.053-1.306
	Longer during weekday & Study ≥ Other causes	0.995	0.906-1.092	1.000	1.205^*^	1.106-1.314	0.948	0.882-1.019	1.000	1.116^*^	1.051-1.185
	Longer during weekend & Study < Other causes	0.941	0.856-1.034	1.000	0.971	0.878-1.074	0.977	0.871-1.095	1.000	1.036	0.951-1.129
	Longer during weekend & Study ≥ Other causes	0.977	0.852-1.121	1.000	1.206^*^	1.054-1.379	0.884	0.774-1.010	1.000	1.048	0.941-1.167

Adjusted for Age, Living with Parents, Economic status, Grade, Alcohol status, Smoking status, Physical activity, Perceived stress level, Self-report Health status, Sleep duration, year 2020(Coronavirus Disease 2019)

Abbreviations: *OR* Odds ratio, *CI* Confidence intervals

^*^Statistically significant

## Discussions

This study was conducted to identify whether sedentary behavior is associated with depressed mood among Korean adolescents based on self-report data. Essentially, sedentary behavior is divided into two categories: weekday or weekend and due to studying or non-studying with covariate adjustment. Additionally, we investigated whether sedentary behavior is associated with suicidality among depressive adolescents.

Previous studies on the relationship between sedentary behavior and depression show different results. According to a review article about the relationship between depression and screen time (ST), such as watching television and playing computer games, the results showed that ST is associated with a 12.3% higher risk of depression among preadolescent children and adolescents [22]. Specifically, for teenagers below 14 years old, two hours or more of ST and only playing computer games are associated with depression [22]. In another review article published in 2016 and a cross sectional study involving low- and middle-income countries, there was strong evidence for the positive association between depressive symptoms and ST for leisure, such as television viewing, electronic gaming, and the use of computers [23, 24]. According to a review article published in 2015, watching television had the largest effect on depression [25]. However, in another review article [26], albeit not focused on adolescents, mentally active sedentary behavior, such as reading, using a computer, meeting and driving, is not associated with depression. However, mentally passive sedentary behavior, such as watching television, listening, talking, and idling can increase the prevalence of depression.

Our hypothesis was that longer total sedentary time, especially longer studying time is positively associated with the prevalence of depressed mood among Korean adolescents. As shown in Table 3, ‘Study ≥ Other causes’ in HS subgroups results supported this hypothesis except in ‘Longer during weekend & Study ≥ Other causes’ subgroup among females. Also, this tendency was consistent with Table 4, regardless of suicidality. On the other hand, students with longer sedentary time during weekday were positively associated with depressed mood with suicidality regardless of the cause of sedentary behavior (Table 4). It would be supposed that targeting the students who spend longer total sedentary behavior, especially longer during weekday is an additional screening option for depressive students with suicidal risk.

Among male students who spend additional sedentary time during weekdays and additional sedentary time for non-studying, LS is positively associated with depression unlike other subgroups (Table 3). However, weekday sedentary behavior usually involves attending classes among Korean students. Moreover, this subgroup had spent only 1.4 hours for studying during weekends which is shorter than other subgroups (not shown in the paper). Therefore, it is also necessary to evaluate whether male students who spend additional time on non-studying activities during weekday have other obstacle factors for concentrating in class such as familial, interpersonal, or psychological problems.

Among females in LS, spending additional sedentary time during weekends and additional time on non-studying activities was determined to be a protective factor for depressed mood especially with no suicidality (Tables 3 and 4). It can be suggested that decreasing total sedentary duration with increasing sedentary behavior for resting or leisure rather than studying could be an option of intervention for depressive students who do not want or unable to follow other well-known intervention.

The mechanism of association between depressed mood and sedentary behavior must be researched further. In previous studies, some plausible explanations are deduced. Increased sedentary time means reduced physical activity, which is a protective factor of depressed mood [27]. Additionally, increased sedentary time would interrupt social relationships and cause depressed mood [28]. Sedentary behavior can cause unfavorable health statuses, including cardiometabolic risk factors [29], pain [30], loneliness [31], and it might be associated with depressed mood. In the aspect of biology, sedentary behavior causes the increase of C-Reactive Protein (CRP), which is an inflammatory factor [32], and finally, it might result in the increased potential of depressed mood [33]. Looking at the study results, we can suggest another main mechanism between sedentary behavior and depressed mood. It could be supposed that distressful and unstructured sedentary behavior, such as studying during weekends, would affect the prevalence of depressed mood. Therefore, increased sedentary time during weekends owing to studying would be a good risk factor of depressed mood.

This study has several limitations. Because of the characteristics of the cross-sectional data, we cannot confirm the cause-effect conclusion, only the association between sedentary behavior and the prevalence of depressed mood. Additionally, KYRBS is a self-report survey, and thus, the accuracy of data would be declined. For instance, unlike clinical interviews conducted by experts, the experience of depressed mood was evaluated using one survey question and the analysis based on divided suicidality such as suicidal ideation, plan and attempt was not included. Therefore, there was no clinical diagnosis and severity assessment for depressive disorder. There was also no objective measurement for sedentary behavior and sleep quality. At the same time, the only criteria for evaluating sedentary behavior were studying or non-studying. Therefore, we could not investigate the association between the detailed cause of sedentary behavior and depressed mood. Furthermore, studying in school during classes, private educational institutes, and independent studying were not included as criteria.

In conclusion, the positive association between the prevalence of depressed mood and sedentary behavior was shown in this study. Further studies based on prospective designs to establish the causality of depressed mood are required. The effects of interventions targeting sedentary behavior or the accuracy of inadequate or excessive sedentary behavior as predictors of depressed mood with or without suicide should be researched.

## Data Availability

The data analyzed in this study were taken from the 2014–2020 KYRBS which is available to the public. All data can be downloaded from the KYRBS official website (https://www.kdca.go.kr/yhs/).
